# Microbiome Associations of Therapeutic Enteral Nutrition

**DOI:** 10.3390/nu6115298

**Published:** 2014-11-21

**Authors:** Rajesh Shah, Richard Kellermayer

**Affiliations:** 1 Department of Internal Medicine, Section of Gastroenterology, Baylor College of Medicine, Houston, TX, 77030, USA; E-Mail: rajeshs@bcm.edu; 2 Department of Pediatrics, Section of Gastroenterology, Baylor College of Medicine and Texas Children’s Hospital, Houston, TX, 77030, USA

**Keywords:** enteral nutrition, Crohn’s disease, microbiota, IBD, rheumatoid arthritis

## Abstract

One of the most effective forms of therapeutic enteral nutrition is designated as “exclusive enteral nutrition” (EEN). EEN constitutes the monotonous enteral delivery of complete liquid nutrition and has been most explored in the treatment Crohn’s disease (CD), a form of inflammatory bowel disease. While EEN’s mechanisms of action are not clearly understood, it has been shown to modify the composition of the intestinal microbiome, an important component of CD pathogenesis. The current literature on the intestinal microbiome in healthy individuals and CD patients is reviewed with respect to EEN therapy. Further investigations in this field are needed to better understand the role and potential for EEN in chronic human disorders.

## 1. Introduction

Therapeutic enteral nutrition describes the provision of defined nutritional support with the goal of treating a disease state. Despite a long history using therapeutic enteral nutrition, the critical mechanisms of action by which dietary changes can exert beneficial effects in specific human disorders are incompletely understood. Multiple hypotheses have been put forward in this regard, which include reduction in antigen exposure, overall nutritional repletion, improvement in intestinal barrier function, provision of micronutrients and reduction in dietary fat or carbohydrate, leading to reduced byproducts producing inflammation [1,2].

Exclusive enteral nutrition (EEN) is a subtype of therapeutic nutrition where dietary intake is confined to the persistent delivery of the same complete food preparation. Most commonly, EEN is achieved by liquid enteral formulas. Liquid formulations of EEN are typically divided into either being elemental or polymeric. Elemental formulas deliver a protein source as individual amino acids, and polymeric formulas provide intact protein. EEN has been most commonly employed to treat Crohn’s disease (CD) [3]. CD is an incurable subtype of inflammatory bowel diseases (IBDs) characterized by transmural inflammation of the intestine anywhere from the mouth to the anus. The pathogenesis of CD is believed to involve the interaction between a defective intestinal barrier, environmental risk factors, the intestinal microbiome and host genetics [4,5]. Many of these biological systems, apart from genetics, are suspected to be modulated by EEN [2], which may explain its efficacy in treating CD. More recently, the microbiome related effects of this therapeutic nutrition are coming into focus. EEN has also been explored in the treatment of other autoimmune diseases, such as juvenile idiopathic arthritis [6] and ulcerative colitis [7], but these studies are rather limited. Therefore, this review will focus on CD with respect to the microbiome-related influences of EEN.

## 2. Treatment of CD with EEN

Induction and maintenance of remission (symptom free disease) are the current treatment goals for CD. The available medical therapies to achieve these goals include 5-aminosalicyclic acid (5-ASA) derivatives, immunosuppressive or immunomodulator agents (azathioprine, methotrexate), biologic medications (molecularly-engineered antibodies against key components of the human intestinal inflammatory cascade: infliximab, adalimumab, certolizumab, natalizumab, vedolizumab, *etc.*) or steroids (prednisone, budesonide). For severe CD, induction is typically achievable with either biologics or steroids. Thereafter, the continuation or transition to immunomodulators or biologic agents can substantiate remission. This approach, however, has limitations, including increased risk of adverse events (pancreatitis, hepatitis, neutropenia/bone marrow suppression) and potential long-term complications, such as a higher risk of malignancies [8,9]. Secondary to the limitations of conventional medical treatments, EEN has been explored as an adjunct or monotherapy for CD.

Since steroids are commonly used to induce remission, studies compared the efficacy of EEN to steroids during early CD treatment. Small, randomized trials found that children treated with elemental nutrition or steroids had similar rates of clinical improvement. Elemental EEN, however, was superior to steroids in terms of improved growth velocity [10,11]. Though these results were encouraging, the treatment durations were only 4–6 weeks, leaving questions about the potential for EEN as a maintenance therapy. Another limitation of these studies was non-compliance, since 8%–11% of children were unable to tolerate the elemental diet due to bad taste.

To overcome the limitations of elemental EEN, some trials have used polymeric formulas to induce or maintain remission in CD patients. Early investigations comparing polymeric formulations to steroids suggested that steroids were superior for inducing remission [12,13]; however, more recent work demonstrated equal efficacy of the two treatments [14,15]. Comparison between these trials is difficult, since they used different formulations of polymeric nutrition and for varied durations. Small cohort studies have examined if patients maintained on EEN (elemental or polymeric) remain in remission, but the results suggested that approximately 40%–60% will relapse within a year [16,17,18]. However, a significant proportion of this relapse rate may be attributed to discontinuation of EEN, which occurred in 20%–50% of the patients. These studies indicate that elemental and polymeric nutrition have equal therapeutic efficacy in CD, so either may be considered. Despite encouraging efficacy results for polymeric nutrition, high relapse rates likely arising from patient non-compliance and deliberate discontinuation due to taste limit EEN as a maintenance therapy for CD.

The discussed studies predominantly used clinical scores (Crohn’s Disease Activity Index, CDAI) to determine treatment efficiency. More recently, the demonstration of intestinal mucosal healing is becoming a favored treatment endpoint [19]. Mucosal healing has been correlated with reduced risk of disease relapse, for example [20]. Steroids do not effectively induce mucosal healing, but several studies have shown that EEN can [21,22,23,24]. Borrelli *et al.* demonstrated in a randomized controlled trial of 37 children with CD that EEN induced mucosal healing in a greater proportion of patients compared to corticosteroids (*p* < 0.001) at 10 weeks. A single small cohort trial observed that polymeric nutrition induced mucosal healing by magnetic resonance enterography imaging [25]. Magnetic resonance enterography (MRE) can noninvasively detect small bowel mucosal damage with a high sensitivity [26,27]. These studies suggest that EEN may be an efficacious therapy for healing mucosal injury related to CD, but definitive conclusions are limited by patients frequently receiving concomitant IBD medications (6-mercaptopurine, mesalamine, *etc.*). Further well-designed studies will be needed to confirm these findings while controlling for other confounding factors.

Based on the above, EEN appears to be as efficacious and perhaps even superior to steroids, for inducing remission in CD. EEN has several other benefits compared to steroids, including improved growth [10,11] and quality of life [28], optimized bone metabolism [29] and induction of mucosal healing [21,22,23]. Another common complication of CD is intestinal stricturing, which can necessitate intestinal resections. The risk of recurrence and subsequent surgeries remains high after resections [30]. Interestingly, a recent prospective cohort study from Japan indicated that even partial (50% caloric requirements) delivery of EEN can significantly reduce the endoscopic (mucosal) recurrence of the disease at the surgical site after a year of surgery [31]. The overall need for re-operation tended to be less as well (*p =* 0.08) in the partially EEN-treated group.

Despite the encouraging findings above, EEN has to be further explored to clearly determine its therapeutic potential as an induction and/or maintenance therapy for CD. The questions of its utility as a mono- *versus* combination therapy and as exclusive *versus* partial nutrition remain to be answered, as well. Community-based data from Spain suggested that physicians are frequently (63%) recommending EEN to CD patients [32], but several barriers (poor long-term compliance, lack of optimized teaching, variable patient acceptance) create practical limitations for exploring the full potential of this promising treatment modality. Additionally, the mechanism of action for EEN would be important to define for the optimization of this practically challenging treatment, which carries a desirable side effect spectrum, compared to other available medical therapies. In the next section, we will review our limited understanding of EEN biology.

## 3. EEN Mechanisms of Action

**Figure 1:**
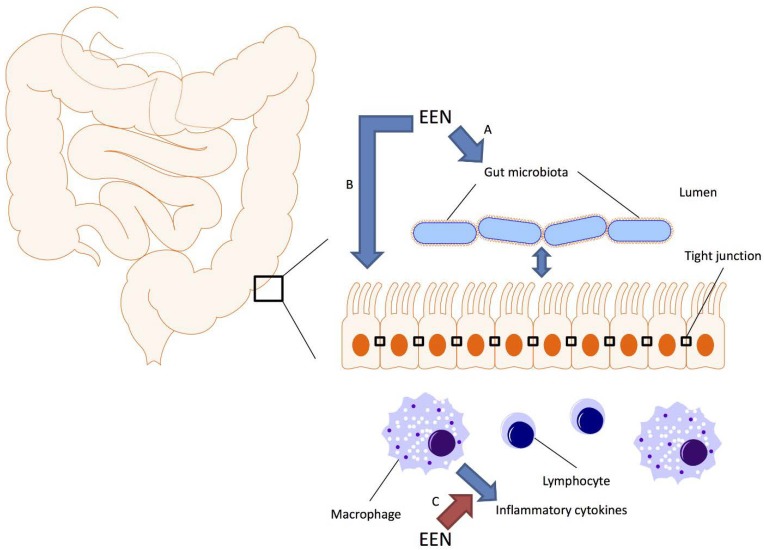
Schematic representation of the gut microbiota, the intestinal epithelium and immune system; and how exclusive enteral nutrition (EEN) may affect these systems. Crohn’s disease is associated with dysbiosis of the gut microbiota, increased permeability of the intestinal epithelium and dysregulated immunity. Studies suggest that EEN can alter the composition of the gut microbiota (A), reduce intestinal permeability through modulation of tight junctions either directly (B) or through modulating the intestinal microbiome (A) and downregulate the production of inflammatory cytokines either directly (C) or through modulating the intestinal microbiome (A).

Several hypotheses have been proposed to explain the efficacy of EEN for CD. The most likely mechanisms include direct anti-inflammatory effects, improvement of intestinal barrier function and modulation of the intestinal microbiome (Figure 1).

CD is characterized by increased production of multiple pro-inflammatory cytokines (TNF-alpha, IL-1, IL-6, *etc.*) [33]. Studies in children have shown that treatment with polymeric enteral nutrition can downregulate mucosal concentrations of these cytokines, as well as improve mucosal integrity [34]. Similar results were found using *in vitro* techniques, where pro-inflammatory signaling pathways were downregulated in the presence of polymeric formula [35,36]. These studies, however, were limited, since a precise molecular mechanism could not be defined, and the authors acknowledged the need for further work in this area. 

Mucosal barrier integrity is vital to the proper functioning of the intestines. Mucosal barrier function is maintained in part by structures designated as tight junctions, which join epithelial cells and participate in the regulation of ion, nutrient and water transport. In CD, the mucosal barrier is dysfunctional, which can be beneficially modified with EEN [37]. *In vitro* models using colonic epithelial cells have demonstrated that EEN can reduce intestinal permeability after the induction of colitis [38,39]. Interestingly, clinical studies have also shown that first-degree family members of CD patients have increased intestinal mucosal permeability when compared to non-IBD controls [40]. These findings suggest that patients with CD have intrinsic mucosal barrier defects, which could be modulated by EEN. However, the exact means by which EEN may improve mucosal permeability are not well understood.

Recent advances in sequencing technologies and bioinformatics have also allowed for examining the potential influence of EEN on the intestinal microbiome, which is another critically important biological system in the pathogenesis of CD. We will next review the human gut microbiome and its potential importance in CD and EEN therapy.

## 4. Microbiome Basics

The human intestinal microbiome comprises a diverse, highly-interactive ecological system of bacteria, Archaea, viruses and fungi [41]. Classically, the intestinal microbiome was characterized by standard microbiological techniques, but those were inherently limited by the inability to culture over 60% of intestinal bacteria. Recently, investigators have leveraged the conserved 16S rRNA gene present in all bacteria to characterize the intestinal microbiome with sequencing techniques. This gene has taxon-specific variable regions, the sequencing of which can allow for bacterial classification. Additional techniques have been developed to sequence all genes present in a tissue sample (whole genome sequencing, WGS) and characterize the overall gene content attributed to microbes [42,43]. These techniques allow for bacterial classification and examination of overall gene content, but lack the ability to provide information about the actual biological activity or function of a specific microbiome. The functional activity of microbiomes is more specifically examined by the maturing fields of metatranscriptomics and metabolomics [42,44].

In parallel with the application of these new sequencing techniques, bioinformatic tools have been developed to process and interpret large datasets [42,45,46]. These tools enable the generation of common ecological measures (richness and diversity) to characterize the intestinal microbiome community. Richness describes the number of unique bacterial (or other microbial) species present in a sample, and diversity accounts for both the number and the relative abundance of the different species. As microbial clinical studies incorporate these metrics, investigators are finding significant associations with multiple diseases, such as obesity [47] and IBD [48].

Results from the American Human Microbiome Project (HMP) and European metagenomics of the human intestinal tract (MetaHIT) projects have provided composition and functional information for the healthy human microbiome [49,50]. These studies showed highly varying intestinal microbiome composition between subjects, but an overall similar functional capacity. From these and subsequent work, we gained an appreciation for the multitude of factors influencing microbiome composition (age, diet, sampling method). Microbiome composition appears to fluctuate rapidly early in life, remain relatively stable during adulthood and change again towards the seventh decade of life [41]. In addition to age, long-term dietary patterns strongly influence the intestinal microbiome [51,52,53]. Additionally, the intestinal microbiome can rapidly respond to short-term dietary interventions, as well, but reverts to its prior composition once the interventions cease [54,55,56]. Furthermore, the gut microbiome is different at the mucosal surface from that of luminal content. Therefore, the clinical sample type (feces *versus* mucosa) has to be taken into consideration [57,58], when planning and comparing microbiome studies of the gut specifically.

## 5. EEN Effects on the Intestinal Microbiome 

A limited number of studies have described the intestinal microbiome of healthy subjects during EEN. Using a cross-over design, Whelan *et al.* examined the effect of fructooligosaccharide and fiber supplemented EEN on intestinal microbiota. They found that total intestinal bacterial counts were reduced during EEN [59]. However, this work utilized fluorescent *in situ* hybridization (FISH) techniques, which carry significant limitations compared to the current high-throughput technologies. Subsequently, the same group re-analyzed this data to describe the change in *Faecalibacterium prausnitzii* during EEN. *F. prausnitzii* is a commensal, Gram-positive bacterium with anti-inflammatory properties, which has been implicated in CD pathogenesis [60]. Their group found significant reductions in *F. prausnitzii* abundance during fiber supplementation, which demonstrated that diet can modulate specific bacterial concentrations [61]. Associations between *F. prausnitzii*, CD and EEN will be discussed in detail in the subsequent sections.

After characterizing the healthy human microbiome by state-of-the-art technology, investigators are now exploring the differences present in diseases. The term dysbiosis refers to the abnormal composition of the intestinal microbiome and has been studied in multiple disease states (obesity, diabetes, *etc.*). Reduced fecal microbiome richness [47] and diversity [62] were found in obese individuals. WGS revealed compositional differences between those with type 2 diabetes and non-diabetic controls, which may influence overall insulin resistance [63,64]. These and many other association studies indicate that abnormal composition and subsequent pathologic functioning of the microbiome may contribute to human disease.

## 6. The Gut Microbiome of Crohn’s Disease

The intestinal microbiome of CD patients has been found to have overall dysbiosis compared to the datasets from HMP and MetaHIT [65,66]. Hansen *et al.* studied colon biopsy specimens from treatment-naive pediatric CD patients and detected significant reductions in diversity compared to healthy controls and patients with ulcerative colitis (UC, the other form of IBD) [65]. Gevers and colleagues examined both stool and mucosal samples from a very large cohort of treatment-naive pediatric CD patients and found modest overall differences compared to controls, which was increased in patients with higher disease activity (Table 1) [66]. Detailed bioinformatic analyses revealed further functional differences between CD and controls. CD microbiomes expressed pathways involved in inflammation and had reduced functionality related to amino acid, carbohydrate and nucleotide metabolism [66]. Other investigations in treatment-experienced CD patients have identified similar functional changes [67,68].

**Table 1:** Description of mucosal microbiota changes found in treatment-naive Crohn’s disease [65,66]. Several bacterial taxa are increased or decreased compared to non-IBD controls. * Changes in *Faecalibacterium* were inconsistent across studies, with one study reporting an increase and the other a decrease.

Crohn’s Disease	
Increased	Decreased
*Enterobacteriaceae*	Bacteroidales
*Pasteurellaceae*	Clostridiales
*Fusobacteriaceae*	*Erysipelotrichaceae*
*Neisseriaceae*	*Bifidobacteriaceae*
*Veillonellaceae*	*Coriobacteriaceae*
*Gemellaceae*	*Faecalibacterium* ^*^
*Faecalibacterium*	

The role of specific bacteria in CD is less understood. The difficulties in making a definite conclusion about single species are well demonstrated by the example of *F. prausnitzii.* Previous observations noted reduced concentrations of *F. prausnitzii* in the terminal ileum of CD patients and higher concentrations correlated with reduced risk of recurrence [69,70]. These studies, however, were confounded by examining treatment-experienced patients, where microbiome composition may have been modified by the treatments themselves [67,71]. Explorations in treatment-naive patients led to conflicting results on *F. prausnitzii*, with one small cohort reporting higher abundance of the bacterium compared to controls [65], as opposed to a lower abundance found in a recent large-scale study [66]. 

These studies emphasize the complexity of the microbiome in IBD and underscore the need for careful planning (*i.e.*, accounting for confounding factors, recruiting adequate number of patients) to enable the detection of meaningful differences. These limitations have to be taken into account when interpreting the available literature on EEN-microbiome effects in CD patients.

## 7. EEN Induced Microbiome Changes in CD Patients

Only a few studies have examined the microbial effects of EEN in CD patients. Lionetti *et al.* reported results from a small case series of nine pediatric CD patients treated with polymeric enteral nutrition. They noted that eight of nine children obtained clinical remission, and all experienced significant shifts in intestinal microbiome composition [72]. These shifts were determined using the gel electrophoresis banding pattern, resulting in a limited depth of interrogation when compared to high-throughput technologies. Similar results were found in a study of six children with CD treated with EEN, as well as correlations between specific bacterial taxa and the degree of inflammation [73]. In a recently published work on EEN treatment for pediatric CD (utilizing gel electrophoresis and quantitative real-time PCR), investigators found decreases in overall microbiome diversity and reductions in *F. prausnitzii* abundance during EEN therapy [74]. These findings challenge the previous notions of increased microbiome diversity associated with health and higher concentrations of *F. prausnitzii* associated with reduced CD activity. In accordance with the difficulties for interpreting the role of *F. prausnitzii* in CD, some observations indicate that regardless of disease activity, the abundance of it remains low in CD [75] and can even further decrease upon clinical improvement with elemental enteral therapy [76]. Clearly, further work is required to understand the role of *F. prausnitzii* in CD and IBD in general. Such controversies emphasize the challenges of microbiome research, even in the current era of advanced technology.

To gain a better understanding of EEN’s effects on the intestinal microbiome, our lab explored this interaction in a mouse model. We set up the hypothesis that it is the monotonous nature of EEN that is critical to its therapeutic effect and not the liquid nature or low antigenicity of it. Healthy mice were assigned to either a single chow or alternating chows. This was meant to mimic either EEN or a more free (liberalized) diet, respectively. After 20 days of the monotonous or the alternating diet feeding, mice were given dextran sulfate sodium (DSS), an intestinal irritant that induces acute large bowel inflammation (colitis) and is an accepted model of human IBD. Mice given the single chow diets, similar to EEN, had higher overall microbiome diversity compared to alternating chow fed mice [77]. These results were consistent with the prior human findings described by Leach *et al.* [73]. However, the results of Gerasimidis and colleagues [74] oppose the observations in the murine experiment. Regardless, the mice provided alternating chow were also more susceptible to intestinal injury from DSS, supporting an association between reduced microbiome diversity and susceptibility to colitis in mammals. This observation is also consistent with the reduction of microbiome diversity in IBD patients [65,66]. Altogether, our murine model work supported the hypothesis that the monotonous nature of EEN may be a key component to its efficacy. Epidemiologic observations correlating agricultural import with the incidence of CD in a European country further substantiated the possible validity of the “monotonous diet” hypothesis [77]. Further high-throughput studies will need to explore the effects of EEN on microbiome composition in both healthy people and CD patients to elucidate the exact effects of this nutritional therapy in health and disease. 

## 8. Future Considerations and Conclusions

Several randomized clinical trials have shown the efficacy for partial and exclusive elemental or polymeric nutritional therapies to induce clinical improvement and even remission in CD. EEN appears to be superior to steroids with respect to the induction of mucosal healing during induction therapy and may have the potential to provide long-term remission in some cases of CD. The highlighted limitations of the existing clinical studies and the practical challenges for this drastic nutritional therapy warrant further intense work towards the optimization of this treatment modality. Though the mechanism(s) of action of nutritional therapy remains unknown, the detected changes in the intestinal microbiome composition and function induced by EEN provide new routes for research. The findings from such investigations may facilitate the development of novel treatment strategies, not only for CD, but for other autoimmune disorders, as well.
